# Thoracoscopic Versus Open Repair of Congenital Diaphragmatic Hernia: A Retrospective Case Series From a Tertiary Care Center

**DOI:** 10.7759/cureus.111125

**Published:** 2026-06-18

**Authors:** Shishir Kumar, Ratan Kumar, Mrunalkant Panchal, Preeti Srivastava, Sanjay K Tanti

**Affiliations:** 1 Department of Surgery, Tata Main Hospital, Jamshedpur, IND; 2 Department of Pediatrics, Tata Main Hospital, Jamshedpur, IND; 3 Department of Pediatrics, Manipal Tata Medical College, Manipal Academy of Higher Education, Jamshedpur, IND

**Keywords:** bochdalek's hernia, congenital diaphragmatic hernia (cdh), minimally invasive surgery, surgical laparotomy, thoracoscopy

## Abstract

Background: Congenital diaphragmatic hernia (CDH) remains a challenging neonatal surgical condition associated with significant morbidity and mortality. Thoracoscopic repair has emerged as a minimally invasive alternative to conventional open repair in selected patients.

Aim: The aim of this study is to share our experience in the surgical management of CDH and to compare postoperative outcomes between thoracoscopic repair and open laparotomy.

Materials and methods: A retrospective analysis of six patients with CDH managed surgically at a tertiary care center was performed. Demographic data, antenatal diagnosis, pulmonary arterial hypertension (PAH), surgical approach, ventilator requirement, initiation of enteral feeding, duration of analgesia, hospital stay, and follow-up outcomes were evaluated.

Results: All patients had left-sided Bochdalek hernias. Three patients underwent thoracoscopic repair, and three underwent open laparotomy. Thoracoscopic repair was associated with a reduced ventilator requirement, earlier initiation of enteral feeds, shorter duration of intravenous analgesia, and reduced hospital stay. One mortality occurred in the laparotomy group. No patient required extracorporeal membrane oxygenation (ECMO) or patch repair.

Conclusion: Thoracoscopic repair of CDH, when performed in carefully selected patients, is safe and associated with improved short-term postoperative outcomes compared to open repair.

## Introduction

Congenital diaphragmatic hernia (CDH) is a rare but severe developmental anomaly characterized by a defect in the diaphragm that permits herniation of abdominal viscera into the thoracic cavity. This results in lung compression, pulmonary hypoplasia, and abnormal pulmonary vascular development. The global incidence of CDH ranges from one in 3,000 to one in 5,000 live births, with substantial variability in survival and long-term morbidity [1,2]. Despite improvements in neonatal intensive care, CDH continues to pose significant therapeutic challenges.

The most common anatomical subtype is the posterolateral Bochdalek hernia, accounting for approximately 90%-95% of cases, with a strong predominance on the left side [2,3]. The severity of disease is primarily determined by the degree of pulmonary hypoplasia and the presence of persistent pulmonary hypertension of the newborn, which represent the principal causes of mortality [4]. CDH is now increasingly recognized as a complex cardiopulmonary disorder rather than a purely surgical condition.

Advances in prenatal ultrasonography and fetal magnetic resonance imaging have improved antenatal detection and risk stratification [5]. Postnatal outcomes have benefited from standardized ventilation strategies, early management of pulmonary hypertension, delayed surgical repair after stabilization, and selective use of extracorporeal membrane oxygenation (ECMO) [6]. Nevertheless, surgical closure of the diaphragmatic defect remains the definitive component of management.

Traditionally, CDH repair has been performed through open laparotomy or thoracotomy, allowing for reduction of herniated viscera and either primary or patch closure of the defect. Over the last two decades, minimally invasive approaches, particularly thoracoscopic repair, have gained popularity. Initially reported in the mid-1990s, thoracoscopic repair offers potential advantages such as reduced surgical trauma, improved visualization of the defect, less postoperative pain, faster recovery, and superior cosmetic results [7,8].

However, concerns persist regarding the safety of thoracoscopic repair, especially in neonates. These include technical complexity, longer operative times, physiological effects of capnothorax leading to hypercapnia and acidosis, and a higher risk of recurrence compared to open repair [9-11]. Most available evidence consists of retrospective studies and meta-analyses, which highlight the importance of patient selection and surgical expertise.

The present study reports our institutional experience with both thoracoscopic and open repair of CDH and compares short-term outcomes between the two approaches, aiming to contribute further evidence to guide surgical decision-making.

## Materials and methods

**Figure 1:**
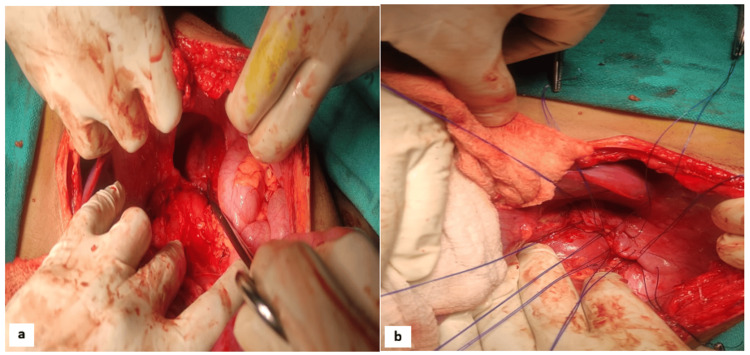
(a) Defect in the left hemidiaphragm; (b) Primary repair of the diaphragm

This retrospective case series included six patients with CDH managed surgically at a tertiary care center. Patient age ranged from three days to nine years. Data were retrieved from hospital records following institutional ethical clearance. Representative intraoperative steps are illustrated in Figure 1.

All patients underwent standardized preoperative stabilization in the neonatal or pediatric intensive care unit. Management included early endotracheal intubation without bag-mask ventilation, echocardiographic evaluation for pulmonary arterial hypertension (PAH), and ultrasonographic assessment of the abdomen, cranium, and spine to identify associated anomalies.

Pulmonary hypertension, when present, was managed medically using sildenafil and/or milrinone. Mechanical ventilation strategies included assist-control pressure-regulated volume control ventilation, with high-frequency oscillatory ventilation used as rescue therapy when required. None of the patients required ECMO support.

**Figure 2:**
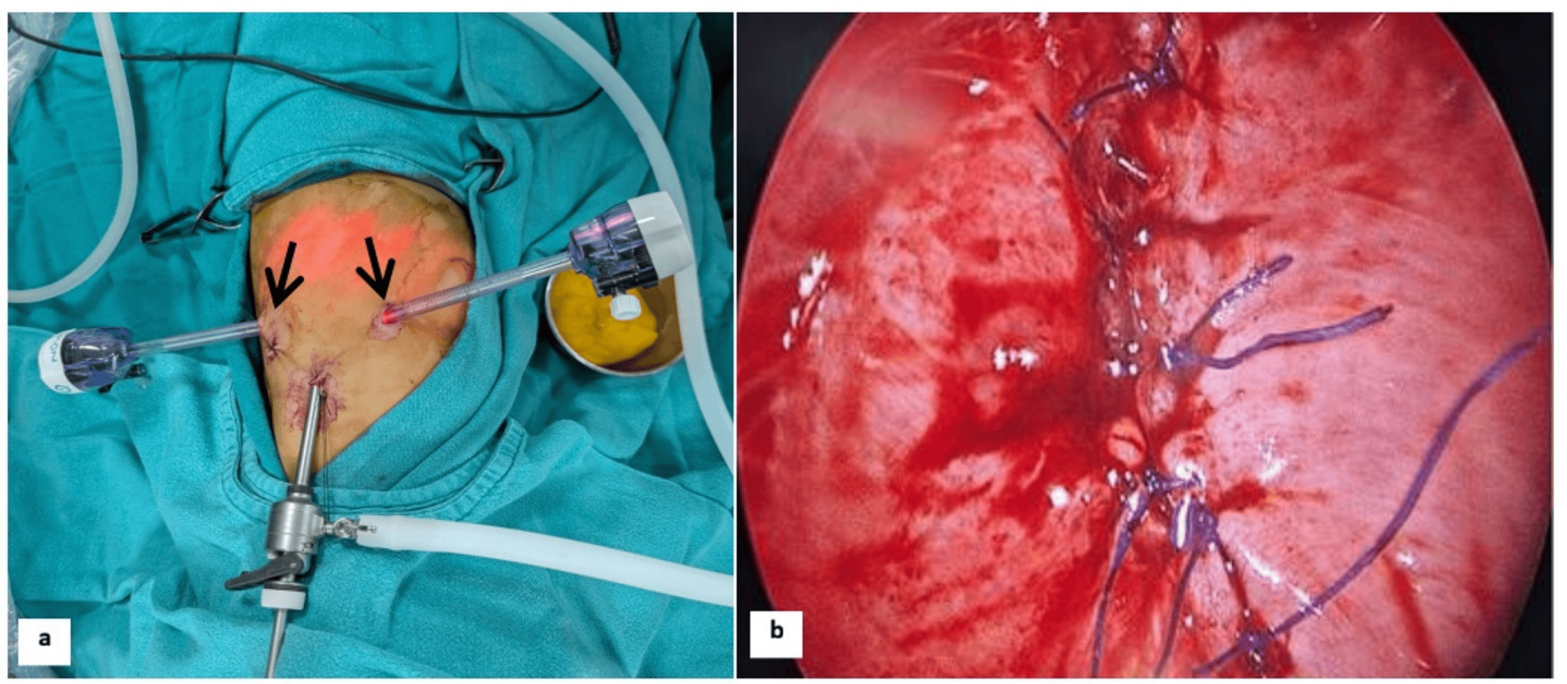
(a) Thoracoscopic port positions (marked by black arrows); (b) Thoracoscopic repair of diaphragm completed

Following stabilization, surgical repair was performed either via conventional open laparotomy or a thoracoscopic approach, based on patient stability and surgeon discretion. Reduction of herniated abdominal contents was followed by primary closure of the diaphragmatic defect using non-absorbable sutures. No patient required prosthetic patch repair (Figure 2).

**Figure 3:**
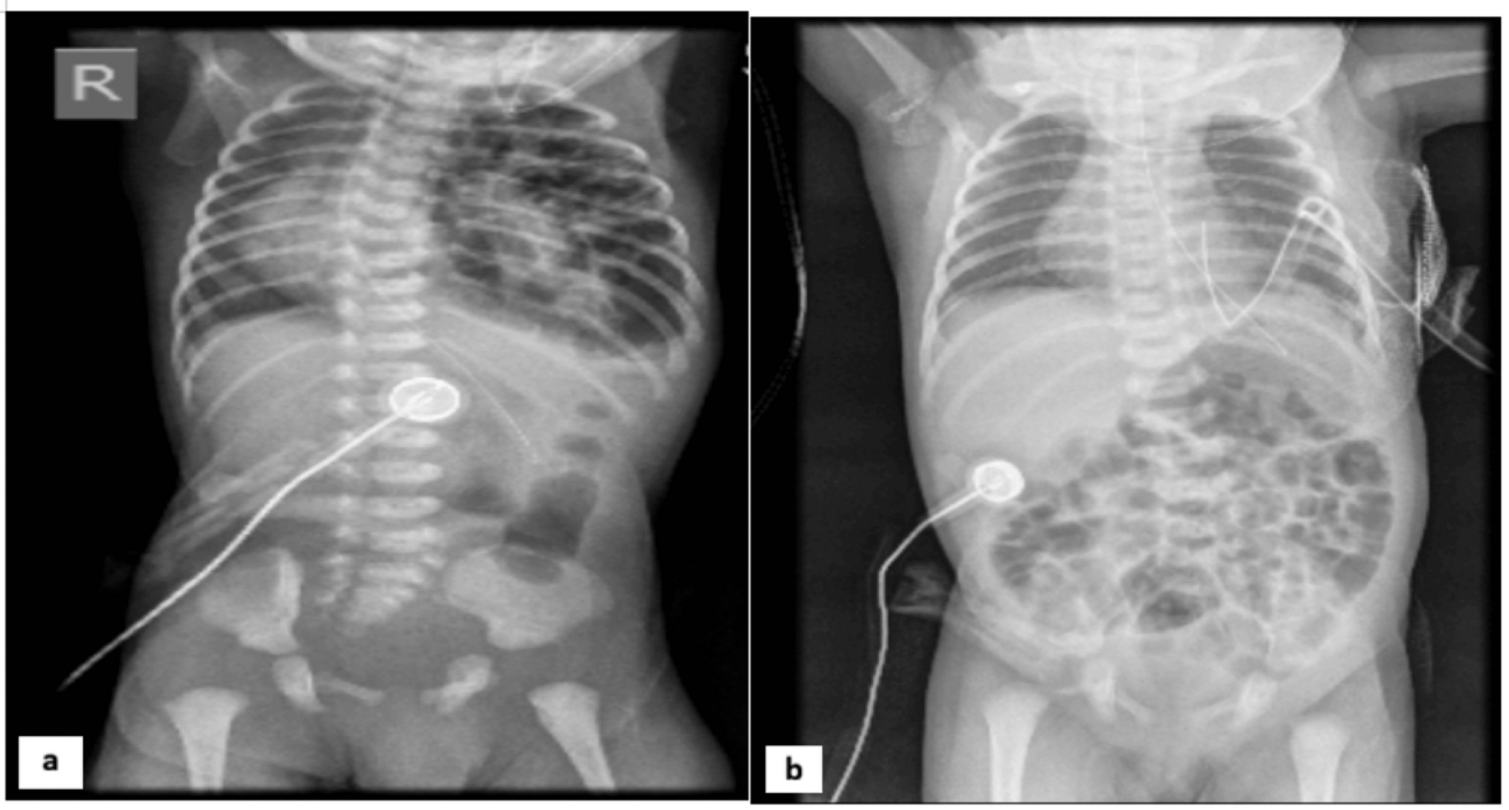
(a) Chest x ray showing herniation of the bowel into the left hemithorax; (b) Postoperative chest x-ray showing complete expansion of the left lung

Postoperative variables assessed included duration of mechanical ventilation, length of intravenous analgesia, time to initiation of enteral feeding, duration of hospital stay, mortality, and recurrence. Lung expansion was checked by postoperative chest x-ray (Figure 3). Follow-up ranged from three months to two years.

## Results

A total of six patients were included in the study. All had left-sided Bochdalek hernia. Antenatal diagnosis was made in three patients. PAH was documented in three neonates at presentation.

Three patients underwent thoracoscopic repair, and three underwent open laparotomy. All defects were closed primarily without the use of a patch. There were no intraoperative complications in either group.

Patients in the thoracoscopic group demonstrated superior short-term outcomes. The mean duration of postoperative mechanical ventilation was 23 hours in the thoracoscopic group compared to 48 hours in the open group. Enteral feeding was initiated significantly earlier following thoracoscopic repair compared to laparotomy. Duration of intravenous analgesia was shorter, and the mean hospital stay was markedly reduced. The comparative outcomes of both groups are tabulated in Table 1.

**Table 1:** Comparative features of six cases of congenital diaphragmatic hernia (CDH)

Case no.	Age	Antenatal Diagnosis	Pulmonary Artery Hypertension	Procedure	Duration of Ventilator Support (Postoperative)	Hospital Stay	Initiation of Enteral Feed (Postoperative Day)	Follow-Up (Months)
1.	9 days	Yes	Moderate	Laparotomy	24 hours	3 days	--	Death
2.	3 days	Yes	Mild	Laparotomy	96 hours	29 days	5	24
3.	3 days	No	Mild	Thoracoscopy	48 hours	5 days	1	12
4.	9 years	No	No	Laparotomy	24 hours	14 days	5	18
5.	2 years	No	No	Thoracoscopy	16 hours	5 days	1	12
6.	2 months	No	No	Thoracoscopy	6 hours	7 days	1	3

One mortality occurred in the open repair group due to significant pulmonary hypertension and respiratory failure. There were no deaths in the thoracoscopic group. During follow-up, no recurrences were reported in either group.

## Discussion

The surgical management of CDH has evolved considerably, driven by improvements in neonatal care and advances in minimally invasive surgery. Our experience demonstrates that thoracoscopic repair, when performed in carefully selected patients, is associated with improved short-term postoperative outcomes compared to open laparotomy.

The reduced duration of mechanical ventilation observed in the thoracoscopic group aligns with findings from multiple meta-analyses reporting decreased ventilator dependency following minimally invasive repair [9,12]. Minimizing ventilator exposure is particularly important in CDH patients, as prolonged mechanical ventilation is associated with ventilator-induced lung injury and adverse neurodevelopmental outcomes.

Early initiation of enteral feeding and shorter requirement for analgesia in our thoracoscopic cohort are consistent with previous studies demonstrating reduced postoperative ileus and pain following minimally invasive surgery [8,13]. These benefits likely result from reduced bowel manipulation and avoidance of large abdominal incisions.

While thoracoscopic repair has been associated with higher recurrence rates in the literature, particularly in larger defects or when patch repair is required, no recurrences were observed in our series [9-11]. This favorable outcome may be attributed to strict patient selection, small defect size, and primary suture repair. Current evidence emphasizes that thoracoscopic repair should be reserved for physiologically stable patients with small to moderate defects [14].

Open repair remains the preferred approach for critically ill neonates, patients with severe pulmonary hypertension, or those with large diaphragmatic defects. Nevertheless, the significantly shorter hospital stay associated with thoracoscopic repair has important implications, particularly in resource-limited settings.

The limitations of the present study include the small sample size, retrospective design, and lack of long-term functional outcomes. Larger prospective studies with standardized selection criteria are required to better define the role of thoracoscopic repair in CDH. 

## Conclusions

Thoracoscopic repair of CDH is a safe and effective alternative to open surgery in carefully selected patients. It offers improved short-term postoperative outcomes, including reduced ventilator requirement, earlier feeding, and shorter hospital stay. A multidisciplinary approach and meticulous patient selection are essential to optimize outcomes.
